# Glances at the Hospitals

**Published:** 1905-09-23

**Authors:** 


### GLANCES AT THE HOSPITALS.

### DUNDEE ROYAL INFIRMARY.

The ordinary income amounted to £11,273 and the ordinary expenditure to £12,038. The extraordinary expenditure was £4,955, and this sum was spent on new sanitary annexes, kitchen appliances, and boiler. Legacies were received during the year amounting to £4,564. At the beginning of the year 227 patients were under treatment, and at the end of it there were 223 in residence. The total number under treatment was 3,540, and of this number 3,317 were treated to a conclusion. Of the total number 2,794 were discharged cured or relieved, 239 not relieved, and 284 died. The highest number in the house on any one day was 261, and the lowest was 215. The average of each patient's stay in the hospital was nearly 24 days. The cost per bed occupied was £50 7s. 5d., which was a few shillings lower than the previous year. The daily average number of nurses in the infirmary was 64, and the daily average number of domestic servants was 39. The medical and surgical tables are extremely well compiled, and convey an unusual amount of information in a manner which deserves praise from us and imitation by those hospitals in which the statistics are so meagre as to be almost without value. During the year there were 71 cases of rheumatic fever in the wards, and of these 70 recovered, with an average duration of 26 days' treatment, and 1 died. Of 33 cases of diphtheria 5 died. Of 50 cases of enteric fever 6 died. There is an anæsthetic table which shows that chloroform was given 1,382 times, ether 9, chloroform and ether 3, chloroform and A.C.E. 3, A.C.E. 8, ether and nitrous oxide 4, ethyl chloride 58, and nitrous oxide 104.

